# Supplementary material for: The relationship between low energy availability, injuries, and bone health in recreational female athletes
Source: PeerJ. 2024 Jun 21;12:e17533. doi: 10.7717/peerj.17533 (PMC11195543; doi:10.7717/peerj.17533)
Supplement: Supplemental Information 2 [file peerj-12-17533-s002.docx]

**Table S1**: Results of normality testing for analysed variables when divided by the LEAF-Q score.

| **Variable** | **Group** | **Shapiro-Wilk** | ***p*** |
| --- | --- | --- | --- |
| LEAF-Q | Non-Risk Group | .8865 | .106 |
| LEAF-Q | Risk Group | .9457 | .575 |
| Body weight fluctuations (kg) | Non-Risk Group | .7571 | **.003** |
| Body weight fluctuations (kg) | Risk Group | .911 | .220 |
| BMD total body (g/cm2) | Non-Risk Group | .9505 | .644 |
| BMD total body (g/cm2) | Risk Group | .9382 | .475 |
| BMD L spine (g/cm2) | Non-Risk Group | .9039 | .178 |
| BMD L spine (g/cm2) | Risk Group | .9492 | .625 |
| BMD neck (g/cm2) | Non-Risk Group | .9293 | .373 |
| BMD neck (g/cm2) | Risk Group | .9004 | .161 |
| Fat free mass (kg) | Non-Risk Group | .9008 | .163 |
| Fat free mass (kg) | Risk Group | .9588 | .766 |
| Body mass (kg) | Non-Risk Group | .7956 | .008 |
| Body mass (kg) | Risk Group | .9637 | .836 |
| Fat mass (%) | Non-Risk Group | .8767 | .079 |
| Fat mass (%) | Risk Group | .8612 | .051 |
| EO COP average velocity. mm/sec | Non-Risk Group | .7891 | **.007** |
| EO COP average velocity. mm/sec | Risk Group | .9414 | .517 |
| EC COP average velocity. mm/sec | Non-Risk Group | .8831 | .096 |
| EC COP average velocity. mm/sec | Risk Group | .9508 | .648 |

**Bold** = Non-Normal Distribution

**Table S2**: Results of normality testing for analysed variables when divided by the occurrence of injury in the past year.

| **Variable** | **Injury in the past year** | **Shapiro-Wilk** | ***p*** |
| --- | --- | --- | --- |
| LEAF-Q | No | .6976 | **< .001** |
| LEAF-Q | Yes | .9631 | .830 |
| Body weight fluctuations (kg) | No | .8036 | **.004** |
| Body weight fluctuations (kg) | Yes | .7689 | **.009** |
| BMD total body (g/cm2) | No | .9405 | .389 |
| BMD total body (g/cm2) | Yes | .9816 | .972 |
| BMD L spine (g/cm2) | No | .9512 | .544 |
| BMD L spine (g/cm2) | Yes | .9687 | .883 |
| BMD neck (g/cm2) | No | .9273 | .249 |
| BMD neck (g/cm2) | Yes | .9535 | .728 |
| Fat free mass (kg) | No | .9166 | .171 |
| Fat free mass (kg) | Yes | .9865 | .989 |
| Body mass (kg) | No | .8297 | **.009** |
| Body mass (kg) | Yes | .9395 | .577 |
| Fat mass (%) | No | .8651 | .029 |
| Fat mass (%) | Yes | .8496 | .074 |
| EO COP average velocity. mm/sec | No | .7935 | **.003** |
| EO COP average velocity. mm/sec | Yes | .9373 | .554 |
| EC COP average velocity. mm/sec | No | .8773 | **.043** |
| EC COP average velocity. mm/sec | Yes | .8861 | .182 |

**Bold** = Non-Normal Distribution

**Table S3**: Results of normality testing for analysed variables when divided by the age of menarche.

| **Variable** | **Age of menarche** | **Shapiro-Wilk** | ***p*** |
| --- | --- | --- | --- |
| LEAF-Q | Up to 15 | .8321 | **.004** |
| LEAF-Q | 15+ | .7765 | .066 |
| Body weight fluctuations (kg) | Up to 15 | .8771 | **.019** |
| Body weight fluctuations (kg) | 15+ | .7907 | .087 |
| BMD total body (g/cm2) | Up to 15 | .9711 | .798 |
| BMD total body (g/cm2) | 15+ | .8172 | .137 |
| BMD L spine (g/cm2) | Up to 15 | .9721 | .818 |
| BMD L spine (g/cm2) | 15+ | .7769 | .067 |
| BMD neck (g/cm2) | Up to 15 | .9557 | .491 |
| BMD neck (g/cm2) | 15+ | .7956 | .095 |
| Fat free mass (kg) | Up to 15 | .9491 | .381 |
| Fat free mass (kg) | 15+ | .9514 | .725 |
| Body mass (kg) | Up to 15 | .8751 | **.018** |
| Body mass (kg) | 15+ | .9575 | .763 |
| Fat mass (%) | Up to 15 | .9206 | .116 |
| Fat mass (%) | 15+ | .9547 | .746 |
| EO COP average velocity. mm/sec | Up to 15 | .7531 | **< .001** |
| EO COP average velocity. mm/sec | 15+ | .9747 | .870 |
| EC COP average velocity. mm/sec | Up to 15 | .8516 | **.007** |
| EC COP average velocity. mm/sec | 15+ | .9938 | .976 |

**Bold** = Non-Normal Distribution

**Table S4**: Results of normality testing for analysed variables when divided by the perceived menstrual bleeding changes related to training.

| **Variable** | **Menstrual bleeding changes related to training** | **Shapiro-Wilk** | ***p*** |
| --- | --- | --- | --- |
| LEAF-Q | No | .6108 | **< .001** |
| LEAF-Q | Yes | .8731 | .085 |
| Body weight fluctuations (kg) | No | .8812 | .074 |
| Body weight fluctuations (kg) | Yes | .8862 | .125 |
| BMD total body (g/cm2) | No | .9467 | .549 |
| BMD total body (g/cm2) | Yes | .8874 | .129 |
| BMD L spine (g/cm2) | No | .9069 | .166 |
| BMD L spine (g/cm2) | Yes | .9484 | .623 |
| BMD neck (g/cm2) | No | .926 | .302 |
| BMD neck (g/cm2) | Yes | .8623 | .062 |
| Fat free mass (kg) | No | .8871 | .089 |
| Fat free mass (kg) | Yes | .9579 | .745 |
| Body mass (kg) | No | .7193 | **< .001** |
| Body mass (kg) | Yes | .9723 | .909 |
| Fat mass (%) | No | .8398 | **.021** |
| Fat mass (%) | Yes | .8833 | .115 |
| EO COP average velocity. mm/sec | No | .7646 | **.003** |
| EO COP average velocity. mm/sec | Yes | .9243 | .356 |
| EC COP average velocity. mm/sec | No | .8801 | .072 |
| EC COP average velocity. mm/sec | Yes | .955 | .708 |

**Bold** = Non-Normal Distribution

**Table S5**: Results of normality testing for analysed variables when divided by experience with menstrual disfunction.

| **Variable** | **Menstrual dysfunction** | **Shapiro-Wilk** | ***p*** |
| --- | --- | --- | --- |
| LEAF-Q | No | .8004 | **.004** |
| LEAF-Q | Yes | .8379 | .055 |
| Body weight fluctuations (kg) | No | .748 | **< .001** |
| Body weight fluctuations (kg) | Yes | .9329 | .510 |
| BMD total body (g/cm2) | No | .936 | .335 |
| BMD total body (g/cm2) | Yes | .9655 | .853 |
| BMD L spine (g/cm2) | No | .9676 | .820 |
| BMD L spine (g/cm2) | Yes | .9525 | .718 |
| BMD neck (g/cm2) | No | .9754 | .928 |
| BMD neck (g/cm2) | Yes | .9063 | .291 |
| Fat free mass (kg) | No | .9283 | .258 |
| Fat free mass (kg) | Yes | .9359 | .539 |
| Body mass (kg) | No | .7892 | **.003** |
| Body mass (kg) | Yes | .9056 | .286 |
| Fat mass (%) | No | .9254 | .233 |
| Fat mass (%) | Yes | .9295 | .477 |
| EO COP average velocity. mm/sec | No | .7427 | **< .001** |
| EO COP average velocity. mm/sec | Yes | .8769 | .146 |
| EC COP average velocity. mm/sec | No | .8872 | .061 |
| EC COP average velocity. mm/sec | Yes | .831 | **.046** |

**Bold** = Non-Normal Distribution
